# Air Pollution and Stillbirth Risk: Exposure to Airborne Particulate Matter during Pregnancy Is Associated with Fetal Death

**DOI:** 10.1371/journal.pone.0120594

**Published:** 2015-03-20

**Authors:** Emily DeFranco, Eric Hall, Monir Hossain, Aimin Chen, Erin N. Haynes, David Jones, Sheng Ren, Long Lu, Louis Muglia

**Affiliations:** 1 Perinatal Institute, Cincinnati Children’s Hospital Medical Center, Cincinnati, Ohio, United States of America; 2 Division of Maternal-Fetal Medicine, Department of Obstetrics and Gynecology, University of Cincinnati College of Medicine, Cincinnati, Ohio, United States of America; 3 Division of Biostatistics and Epidemiology, Cincinnati Children’s Hospital Medical Center, Cincinnati, Ohio, United States of America; 4 Department of Environmental Health, University of Cincinnati College of Medicine, Cincinnati, Ohio, United States of America; 5 Department of Mathematics, University of Cincinnati, Cincinnati, Ohio, United States of America; 6 Division of Biomedical Informatics, Cincinnati Children’s Hospital Medical Center, Cincinnati, Ohio, United States of America; The Ohio State University, UNITED STATES

## Abstract

**Objective:**

To test the hypothesis that exposure to fine particulate air pollution (PM_2.5_) is associated with stillbirth.

**Study Design:**

Geo-spatial population-based cohort study using Ohio birth records (2006-2010) and local measures of PM_2.5_, recorded by the EPA (2005-2010) via 57 monitoring stations across Ohio. Geographic coordinates of the mother’s residence for each birth were linked to the nearest PM_2.5_ monitoring station and monthly exposure averages calculated. The association between stillbirth and increased PM_2.5_ levels was estimated, with adjustment for maternal age, race, education level, quantity of prenatal care, smoking, and season of conception.

**Results:**

There were 349,188 live births and 1,848 stillbirths of non-anomalous singletons (20-42 weeks) with residence ≤10 km of a monitor station in Ohio during the study period. The mean PM_2.5_ level in Ohio was 13.3 μg/m^3^ [±1.8 SD, IQR(Q1: 12.1, Q3: 14.4, IQR: 2.3)], higher than the current EPA standard of 12 μg/m^3^. High average PM_2.5_ exposure through pregnancy was not associated with a significant increase in stillbirth risk, _adj_OR 1.21(95% CI 0.96,1.53), nor was it increased with high exposure in the 1^st^ or 2^nd^ trimester. However, exposure to high levels of PM_2.5_ in the third trimester of pregnancy was associated with 42% increased stillbirth risk, _adj_OR 1.42(1.06,1.91).

**Conclusions:**

Exposure to high levels of fine particulate air pollution in the third trimester of pregnancy is associated with increased stillbirth risk. Although the risk increase associated with high PM_2.5_ levels is modest, the potential impact on overall stillbirth rates could be robust as all pregnant women are potentially at risk.

## Introduction

The stillbirth rate (fetal death ≥20 weeks of gestation) is higher in the US compared to many developed countries. [1,2] The US stillbirth rate in 2006 was 6.0 per 1000 births,[3] almost 50% higher than the Healthy People 2010 goal of 4.1 per 1000.[4] The rate is higher in Ohio (6.2 per 1000) and further increased in the city of Cincinnati (6.9 per 1000), one of the most populous areas of the state. A variety of pre-existing medical, socioeconomic, prenatal, genetic, and environmental factors influence a woman’s individual risk of stillbirth.

Exposure to harmful environmental pollutants is associated with adverse health outcomes and pregnancy complications.[5,6] Airborne particulate matter (PM) is a complex mixture of extremely small particles and liquid droplets including acids, organic chemicals, metals, and soil or dust particles. The smallest particles easily pass into the lungs and have the highest potential to negatively affect multiple organ systems. PM_2.5_, fine particulate matter, measures <2.5 μm in aerodynamic diameter. The US Environmental Protection Agency (EPA) has air quality standards for particle pollution, and monitors local levels via stationary monitoring stations throughout the US. The US EPA National Ambient Air Quality Standard (NAAQS) for annual mean level of PM_2.5_ is currently 12 μg/m^3^.[7]

Few prior studies have reported the association between air pollutants and stillbirth,[8–13] and only two have assessed high exposure to PM_2.5_ specifically and reported no significant association.[14,15] Previous studies have been limited in design by exposures with measures at a single time point or with a lack of geographic granularity, and lack thorough adjustment for important clinical or socio-demographic risk factors. In this study we aim to integrate air quality measures from statewide monitoring stations with vital records to perform geospatial analyses testing the hypothesis that exposure to fine particles in the air (PM_2.5_) is associated with stillbirth risk.

## Materials and Methods

### Study design

Geo-spatial population-based cohort study using Ohio state birth records (2006–2010) and local measures of PM_2.5_, recorded by the US Environmental Protection Agency (2005–2010) via 57 monitoring stations across Ohio.

### Exposure

The primary exposure was high level of airborne PM_2.5_, fine particulate matter in the air measuring <2.5 μm in diameter. High exposure was defined as ≥ mean PM_2.5_ level plus interquartile range for the specific time period measured for each birth.

### Outcome

The primary outcome was stillbirth. Stillbirth is recorded through vital records in the US using the definition of fetal death per the World Health Organization: birth of a fetus with no evidence of life such as movement, breathing, or heartbeat, irrespective of the duration of gestation and which is not an induced termination of pregnancy.[16] As is commonly recommended, we considered only stillbirths with birth weight ≥350 grams and occurring at ≥20 weeks of gestation for this analysis.[17] The reference group for comparison was comprised of live births also with birth weight ≥350 grams and occurring at ≥20 weeks of gestation. Gestational age was defined by the best obstetric estimate variable in the birth record, which combines last menstrual period and ultrasound parameters, as is commonly accepted in clinical practice for gestational age estimation.

### Study population

All live births and stillbirths that occurred in Ohio during the 5 year study period, 2006–2010. The study cohort was limited to singleton births occurring at 20–42 weeks of gestation without known major congenital anomalies, and with mother’s residence within 10 km of a PM_2.5_ monitoring station. All live birth and stillbirth data were obtained from the Ohio Department of Health vital statistics database.

### Statistical analyses

Daily measures of PM_2.5_, recorded by 57 monitoring stations across the state of Ohio, were obtained from the Environmental Protection Agency from 2005–2010^18^ and monthly averages of PM_2.5_ were calculated for each station. Using geographic coordinates of the mother’s residence for each birth and ArcGIS 10.1 (ESRI, Redlands, CA) software, vital records were linked to data from the nearest PM_2.5_ monitoring station. Average PM_2.5_ exposure level per trimester was calculated for each birth occurring within 10 km of a monitoring station.

Demographic, medical and delivery characteristics of stillbirths were compared to live births using t-test for continuous variable comparisons and χ^2^ tests for categorical variables. The association between stillbirth risk and high PM_2.5_ levels was estimated using generalized estimating equation (GEE) model with logit link function, with adjustment for maternal age, race, education level, quantity of prenatal care, cigarette smoking—each as recorded in the birth certificate, as well as season of conception which was created from the date of birth and gestational age at birth. There was minimal missing data for maternal residential address (2.3%), less than 0.1% missing maternal age or smoking status, 0.8% missing maternal race, 27.2% missing data prenatal care initiation, and 0.9% missing data on maternal educational level. There was a higher amount of missing data number of cigarettes smoked per day for smokers with stillbirth (31% missing), and therefore the dichotomous smoking status (yes/no) with minimal missing data (<0.01%) was utilized for analyses rather than modeled as a continuous variable. An exchangeable correlation matrix for the monitoring stations was used in the GEE models to account for spatial correlation within the same PM_2.5_ monitor. Analyses were performed for births with residence within 10 km of a monitoring station, and then repeated as a sensitivity analysis limited to those with residence within 5 km of a monitoring station.

Analyses were performed using SAS version 9.3, SAS Institute Inc., Cary, NC, USA. Attributable risk (AR) was calculated using the formula: AR = Ie—Iu, where Ie = incidence of stillbirth in the high exposure group and Iu = incidence of stillbirth in the unexposed group. The population attributable risk (PAR), the reduction in stillbirth incidence that would be observed if the population were unexposed to high PM_2.5_ levels, compared with its current (actual) exposure pattern was calculated as: PAR = Ip—Iu, where Ip is the population incidence. Population attributable risk percentage (PAR%) was calculated as: PAR% = 100 x Pe (RR—1)/(Pe(RR-1)+1), where Pe is percentage of high exposure in the entire population (approximately 10% for the population included in this study) and RR is relative risk.

Stillbirth rates are reported as number of stillbirths per 1000 total births (live births plus stillbirths). Comparisons with a probability value <0.05 or 95% confidence interval without inclusion of the null were considered statistically significant.

The Ohio Department of Health and Human Subjects Institutional Review Board approved the protocol for this study. This study was exempt from review by the Institutional Review Board at the University of Cincinnati, Cincinnati, Ohio. A de-identified data set generated from vital records of all live births and stillbirths that occurred in the state from 2006–2010 was provided for this analysis by the Ohio Department of Health.

## Results

**Fig 1:**
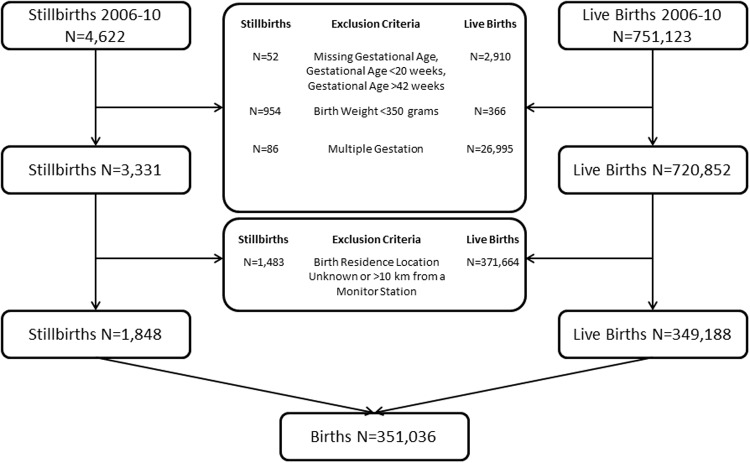
Flow diagram of the study population, Ohio births 2006–2010.

There were 751,123 live births and 4,622 stillbirths in Ohio during the 5 year study period. We focused this analysis on singleton births occurring at 20–42 weeks of gestation without major congenital anomalies. The measurement of association between PM_2.5_ levels and stillbirth was limited to births to mothers whose residence was within 10 km of a PM_2.5_ monitoring station, resulting in a study sample of 351,036 births: 1,848 stillbirths and 349,188 live births, Fig. 1.

In the cohort studied in this analysis, the stillbirth rate decreased during the study period, from 5.7 per 1000 in 2006 to 4.6 per 1000 births in 2010, Table 1. The rate of stillbirth was higher in very urban areas, 5.4 per 1000, compared to residence in any less urban area, 4.3 per 1000. Most births analyzed (98%) occurred in very urban areas, where most monitoring stations are located and exposure levels are likely to be highest. Stillbirth rates were high among the oldest mothers, age ≥40 years, 11.6 per 1000, and non-Hispanic black mothers, 8.6 per 1000, as well as women with lower education level and tobacco use. Women with no prenatal care had the highest rate of stillbirth, 9.4 per 1000. Season of conception had no influence of stillbirth rates.

**Table 1:** Maternal Characteristics, Ohio Birth Cohort 2006–2010.

**Demographic Factors**				
Advanced maternal age				
35–39 years	12.6	10.2	<0.01	6.5
≥ 40 years	2.7	1.2	<0.01	11.6
Race and Ethnicity				
Non Hispanic White	44.8	63.6	<0.01	3.7
Non Hispanic Black	46.8	28.3	<0.01	8.6
Hispanic	5.8	5.3	0.41	5.7
Other	2.7	2.8	0.83	5.1
**Social Behaviors & Socioeconomic Factors**				
Education				
Less than high school	23.4	20.3	<0.01	5.7
High school graduate	38.7	25.5	<0.01	7.5
College education	38.0	54.2	<0.01	3.5
Tobacco use	25.0	19.4	<0.01	6.4
**Prenatal Care Initiation**				
First trimester	78.7	67.9	<0.01	7.5
Second trimester	13.1	23.8	<0.01	3.6
Third trimester	3.1	4.9	<0.01	4.1
No prenatal care	5.0	3.4	<0.01	9.4
**Year of Birth**				
2006	22.2	20.5	0.07	5.7
2007	20.4	20.5	0.98	5.3
2008	20.4	20.2	0.81	5.3
2009	20.1	19.7	0.66	5.4
2010	16.8	19.1	0.01	4.6
**Season**				
Winter	25.1	24.8	0.76	5.3
Spring	23.8	24.6	0.39	5.1
Summer	25.4	25.1	0.75	5.3
Fall	25.8	25.5	0.81	5.3

Dichotomous variables for first 2 columns are presented as percent of total for each characteristic. Stillbirth rate is presented as number of stillbirths per 1000 total births per each characteristic.

Continuous variables are presented as median (IQR) for non-normally distributed data and mean +/- standard deviation for normally distributed data.

Stillbirths occurred more frequently at 20–24 weeks (34.4%), compared to 25–28 weeks (15.1%), 29–32 weeks (13.9%), 33–36 weeks (18.1%) and term ≥37 weeks (18.4%), Table 2. The stillbirth rate was lowest for births at term, 1.1 per 1000. Considering that a major contributor to stillbirth rates is intrapartum death of a previable or periviable fetus born between 20–24 weeks due to preterm labor, the rate of stillbirth in the 20–24 week gestational age period was highest, as expected, 365.7 per 1000.

**Table 2:** Birth Characteristics, Ohio 2006–2010.

**Birth weight, grams**				
350–2499	78.9	7.7	<0.01	48.1
2500–3999	18.7	84.7	<0.01	1.1
≥4000	2.4	7.6	<0.01	1.6
**Gestational age at birth,weeks**				
20–24	34.4	0.3	<0.01	365.7
25–28	15.2	0.6	<0.01	121.7
29–32	13.9	1.2	<0.01	59.0
33–36	18.1	7.3	<0.01	12.9
≥37	18.4	90.7	<0.01	1.1

Dichotomous variables for first 2 columns are presented as percent of total for each category. Stillbirth rate is presented as number of stillbirths per 1000 total births per each category.

The mean PM_2.5_ level during the study period (2006–2010) in Ohio was 13.3 μg/m^3^ [±1.8 μg/m^3^, IQR (Q1: 12.1, Q3: 14.4, IQR: 2.3)], which is higher than The US EPA National Ambient Air Quality Standard (NAAQS) of 12 μg/m^3^.[7] Mean PM_2.5_ level, IQR for the entire cohort per trimester was: 1^st^ trimester 13.67, 3.53 μg/m^3^; 2^nd^ trimester 13.30, 2.96 μg/m^3^; 3^rd^ trimester 13.05, 3.17 μg/m^3^; pregnancy average 13.32, 2.35 μg/m^3^. High PM_2.5_ level was defined as mean + IQR for each group: 1^st^ trimester 17.2 μg/m^3^; 2^nd^ trimester 16.26 μg/m^3^; 3^rd^ trimester 16.22 μg/m^3^; pregnancy average 15.67 μg/m^3^.

The mean PM_2.5_ levels during first trimester of pregnancy were not significantly different for stillbirths compared to the live birth group However, mean PM_2.5_ levels during the second trimester were slightly lower and during the third trimester were significantly higher for the stillbirth group compared to the live birth group, see Table 3. Overall, pregnancy average PM_2.5_ levels for the entire cohort did not significantly differ between stillbirths and live births, 13.40 ± 1.9 μg/m^3^ versus 13.32 ± 1.75 μg/m^3^, p = 0.09. We identified significant correlations between PM_2.5_ and exposure levels of several other airborne measures of pollutants (p<0.001) including nitrogen dioxide, sulfur dioxide, ozone, carbon monoxide and lead.

**Table 3:** PM_2.5_ levels in Ohio 2006–2010, by trimester of exposure in pregnancy.

PM_2.5_ level	Mean (SD)	IQR (Q3, Q1)	Mean (SD)	IQR (Q3, Q1)	
First trimester	13.60 (2.76)	15.04, 11.72	13.67 (2.87)	15.22, 11.69	0.380
Second trimester	13.18 (2.26)	14.63, 11.59	13.30 (2.36)	14.66, 11.70	0.026
Third trimester	13.24 (2.76)	14.95, 11.29	13.05 (2.34)	14.57, 11.40	0.049
Entire pregnancy	13.32 (1.81)	14.44, 12.07	13.32 (1.75)	14.40, 12.05	0.870

PM_2.5_ levels are expressed as mean air concentration in μg/m^3^. SD = standard deviation, IQR = interquartile range (3^rd^, 1^st^ quartile).

**Fig 2:**
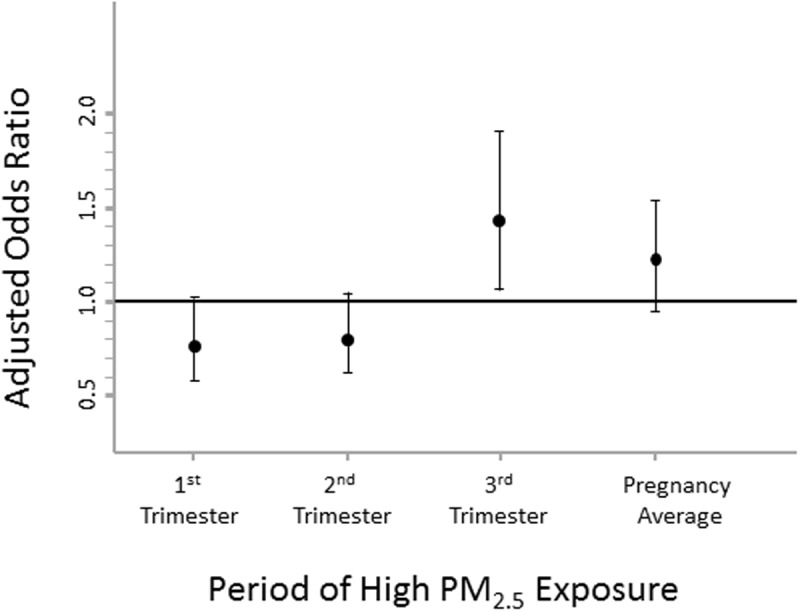
Relative odds of stillbirth associated with exposure to high levels of PM_2.5_, by trimester of pregnancy, Ohio 2006–2010.

When analyzing the outcomes for births within 10 km of a stationary monitor, exposure to high levels of PM_2.5_ throughout the pregnancy was not associated with a significant increase in stillbirth risk, _adj_OR 1.21 (95% CI 0.96, 1.53). Likewise, the risk was not increased with exposure to high PM_2.5_ levels in the 1^st^ trimester, _adj_OR 0.77 (95% CI 0.58, 1.02), or 2^nd^ trimester _adj_OR 0.80 (95% CI 0.62, 1.04). However, exposure to high levels of PM_2.5_ in the third trimester of pregnancy was associated with 42% increased stillbirth risk, _adj_OR 1.42 (95% CI 1.06, 1.91), even after adjustment for important coexisting risk factors for fetal death, Fig. 2. Sensitivity analyses performed using a more strict residential distance-to-monitor cut-off of 5 km demonstrated similar findings. The 5 km cut-off associations demonstrated no difference in stillbirth risk with high PM_2.5_ levels throughout pregnancy or in the second trimester compared to live births. There was minimally decreased stillbirth odds with high 1^st^ trimester exposure. Compared to the 10 km cut-off analysis, there was a slightly more robust effect size of stillbirth odds with high 3^rd^ trimester PM_2.5_ exposure, adjusted OR 1.54 (95% CI 1.08, 2.20), see Table 4. However, if PM_2.5_ exposure was modeled as a continuous variable rather than a dichotomous variable, this finding became non-significant. The relative influence of important coexisting risk factors included in the adjusted analysis of PM_2.5_ exposure and stillbirth risk is depicted in Table 4.

**Table 4:** Logistic regression of factors associated with stillbirth, Ohio 2006–2010.

**Maternal Age, years**		
<20	1.03 (0.80, 1.32)	1.11 (0.85, 1.44)
20–24	0.95 (0.78, 1.15)	0.89 (0.72, 1.10)
25–29	1.00 (Referent)	1.00 (Referent)
30–34	1.11 (0.90, 1.37)	1.00 (0.76, 1.33)
35–39	1.56 (1.25, 1.96)	1.35 (1.07, 1.30)
≥40	2.80 (1.86, 4.21)	2.24 (1.34, 3.75)
**Maternal Race**		
Non-Hispanic white	1.00 (Referent)	1.00 (Referent)
Non-Hispanic black	2.31 (0.99, 5.40)	1.05 (0.46, 2.41)
Hispanic	1.63 (1.04, 2.57)	1.13 (0.57, 2.21)
Other Non-Hispanic	1.39 (0.92, 2.09)	0.87 (0.52, 1.44)
**Maternal Education Level**		
Less than high school	1.77 (1.29, 2.44)	1.77 (1.37, 2.28)
High school only	2.26 (1.89, 2.71)	2.22 (1.76, 2.80)
Postsecondary education	1.00 (Referent)	1.00 (Referent)
**Prenatal Care Initiation**		
First trimester	1.00 (Referent)	1.00 (Referent)
Second trimester	0.34 (0.27, 0.43)	0.39 (0.30, 0.49)
Third trimester	0.32 (0.21, 0.48)	0.30 (0.21, 0.45)
No prenatal care	0.70 (0.40, 1.22)	0.78 (0.49, 1.26)
**Season of conception**		
Winter	1.07 (0.89, 1.29)	1.09 (0.89, 1.32)
Spring	1.00 (Referent)	1.00 (Referent)
Summer	1.13 (0.94, 1.37)	1.15 (0.90, 1.47)
Fall	1.09 (0.91, 1.30)	1.14 (0.98, 1.33)
**Tobacco Use**	1.44 (1.22, 1.71)	1.28 (1.02, 1.62)
**High PM**_**2.5**_ **Exposure**		
Average over pregnancy**	1.21 (0.96, 1.53)	1.06 (0.80, 1.41)
First trimester	0.77 (0.58, 1.02)	0.71 (0.52, 0.96)
Second trimester	0.80 (0.62, 1.04)	0.78 (0.60, 1.02)
Third trimester	1.42 (1.06, 1.91)	1.54 (1.08, 2.20)

*Odds ratio estimates are adjusted for all other factors listed in the first column of the table.

** The odds ratios for all covariates in the table are derived from the logistic regression model of High PM_2.5_ exposure average over pregnancy.

The attributable risk of stillbirth related to high PM_2.5_ exposure (7.48 per 1000 minus 5.2 per 1000) was 2.2 per 1000. The attributable risk percent: (Ie-Iu)/Ie is 30%, i.e. for the exposed population, 30% of stillbirths were from high PM_2.5_ exposure. Assuming 10% of population was exposed to high PM_2.5_, the population attributable risk percentage of high PM_2.5_ exposure is 4% for stillbirth.

## Discussion

### Main findings

In this study we found that exposure to high levels of fine particulate air pollution, PM_2.5_, in the third trimester of pregnancy is associated with increased stillbirth risk. Despite adjustment for important coexisting risk factors for fetal death such as maternal age, race, tobacco use and lack of prenatal care, we found that pregnant women exposed to high levels of PM_2.5_ during the third trimester of pregnancy had a 42% increased risk of stillbirth. This is a novel finding that has not previously been reported.

### Strengths and limitations

One significant limitation of this study, and most studies of air pollution on heath complications, is the approach of measuring quantity of exposure through stationary monitor sites. Although these represent a good estimation of hourly, daily, or longer duration average regional air pollution levels, they do not account for variations in personal exposure levels or amount of variation of indoor air quality. This approach also does not take into consideration variations in pollutant levels throughout the geographic metric included in the analysis, which was a 10 km radius from the monitor station in this study, nor is there any adjustment for possible geographic mobility throughout the pregnancy. However, the rate of residential mobility has been estimated to be fairly low, approximately 12% during pregnancy, and the majority of those who do move stay within the same municipality.[19] There is also the possibility of interaction with other concomitant pollutant exposures or unmeasured sociodemographic and pregnancy risks for stillbirth, which could possibly cluster in areas with high pollutant levels. We identified significant correlations between exposure levels of PM_2.5_ and several other airborne pollutants (p<0.001) including nitrogen dioxide, sulfur dioxide, ozone, carbon monoxide and lead. However, the differing locations of these stationary monitors across the state of Ohio and fewer numbers of monitors for most pollutants compared to PM_2.5_ monitors limited our capacity to accurately assess and adjust for high levels of each other pollutant in women who resided within 5 or 10 km of a stationary PM_2.5_ monitor. Despite this, using individual pollutant-specific stationary monitors to quantify an individual’s air pollution exposure is a commonly used and accepted quality approach of assessing the association between air pollution and birth outcomes.[20–25] Although we did adjust for the most commonly known risk factors for stillbirth, other unmeasured factors could have also influenced our findings and may even explain the positive associations identified, considering the small effect sizes and few significant findings in this study. Other commonly reported limitations of vital statistics data used for research is related to accuracy of the data. Certainly, the choice of variables for analysis affects the study’s internal validity. In this study, stillbirth and live birth are reported in the same way as they are throughout the US, with reliable certainty. Our exposure levels should be reliably estimated as well, as data was obtained directly from daily measures reported by the US EPA.[18] Demographic factors such as maternal age and race are felt to be quite accurate in birth records, and the accuracy of gestational age of birth is considered reliable with a relatively narrow margin of variability in birth certificates as well.[26] Other variables on birth records are known to be underreported, such as ante- and intrapartum complications and co-morbid conditions such as hypertension and diabetes;[27] however, the primary exposure and outcome in this study did not include variables likely to have significant data accuracy concerns. Inability to adjust thoroughly for concomitant medical risk factors could have biased our results toward a positive association, however other design limitations may have biased toward the null.

Our study also has significant strengths compared to other studies on air pollution and pregnancy complications. Our cohort design and time-based approach considering cumulative daily-monthly pollutant levels and assignment to specific time periods of pregnancy by trimester enabled us to assess which time during fetal development may be the most susceptible to high levels of air pollution. In addition, we utilized data on specific location of residence of each birth in the state and were able to assign accurate regional, temporal exposure levels to each live birth and stillbirth. Furthermore, we performed sensitivity analyses by repeating our analyses to births with home residence within a closer proximity to a stationary monitor, 5 km, and demonstrated consistency of effect. The non-significant association of stillbirth risk with high exposure in the third trimester found when analyzing PM_2.5_ as a continuous variable in a sensitivity analysis, differing from the significant association when modeled as a categorical variable in our primary planned analysis, may indicate that there is a threshold above which exposure levels increase risk and may not at mid-range exposure levels, and perhaps not in a linear fashion. Our contemporary study cohort (2006–2010), and large population-based sample size (1,848 stillbirths and 349,188 live births) of US births further improved the external validity and generalizability of our findings. Our study is the largest cohort assessing the influence of PM_2.5_ on stillbirth risk reported to date.

### Interpretation

Differences in reported effects of airborne particulate matter on birth outcomes in the existing literature may be influenced by differences in populations studied.[8,10,12] Some countries have much higher levels of concomitant air pollution density and frequency of other exposures such as maternal tobacco smoking, as in Russia and Greece. In addition, prior study differences may be confounded by geographic location of study, as the composition of particulate matter differs by locale. In China, coal is the major component in particulate air pollution, whereas industrial waste contributes a majority in the Czech Republic, auto exhaust is the major source in South Korea, and dust is the major contributor in the US.[28] Furthermore, prior studies have reported associations with various sizes of airborne particulate matter including total suspended particulates (TSP) and inhalable course particulates <10 μm (PM_10_) which can be found in dusty areas near roadways and some industries. Few studies have investigated fine particulate matter, PM_2.5,_ which is comprised of smoke, haze, and gas emissions. These are the smallest particles in the air with the highest likelihood of being inhaled and subsequently entering the systemic circulation, ultimately causing negative health effects (http://www.epa.gov/airquality/particlepollution/health.html).[5,6]

Only two prior studies have assessed airborne PM_2.5_ influences on stillbirth. Similar to our study, prior investigators (Faiz, et al), estimated the effect of trimester-specific exposures on stillbirth risk.[13] Despite similar study design and also using a population-based sample in the US (New Jersey), but from slightly older time period from 1998–2004, the authors reported a non-significant association between increased PM_2.5_ exposure in the third trimester and stillbirth, adjOR 1.08 (CI 0.79, 1.48). There are several explanations for the differences in study findings. Our study was significantly larger, by more than 50%, compared to the cohort included in the study by Faiz, et al, increasing our statistical power to detect a difference. The direction of effect in Faiz’s study and our study were similar, both demonstrating a modest risk increase; however, our study demonstrated this as a significant finding with 95% confidence interval greater than the null. An additional explanation may be related to their designation of exposure quantity. In their study, stillbirth risk was measured as an interquartile range increase of PM_2.5_ exposure, about 4 μg/m^3^ increase. Our study compared the risk of stillbirth for those women within high PM_2.5_ exposure, >16–17 μg/m^3^, versus those with low exposure levels. Assuming that the highest exposure levels may have the most robust effect on adverse outcome, our choice of exposure characterization may have enhanced our power to detect a difference. A more recent study published by the same authors, Faiz et. al (2013), also found no significant association between increased PM_2.5_ exposure, measured per quartile increase, and stillbirth when quantified as high levels in the immediate few days prior to birth, despite similarly studying a population exposed to high mean levels of PM_2.5_ (mean exposure levels 14.7–15.0 μg/m^3^).[15] Several other studies have analyzed other forms of airborne particulate matter, total suspended particles (TSP) [10] and course particles (PM_10_) [8,12] and found no association with stillbirth, however exposure to larger airborne particles may have a different mechanism of action, or be less influential with regards to negative health effects.[6]

The mechanism by which particulate air pollution might negatively affect the fetus through maternal exposure has not been clearly elucidated. The association between particulate air pollution and poor health outcomes in adults has been more definitively established.[6] In adults, three potential mechanisms of PM exposure have been proposed: stimulation of the inflammatory response resulting in increased coagulation, immune or allergic-type response, and autonomic effects on the cardiovascular system leading to decreased heart rate variability.[5] It is plausible that any or all of these mechanisms may contribute to alterations in uteroplacental perfusion, nutrient and oxygen transfer to the fetus, or even catastrophic thrombotic or ischemic events leading to abrupt fetal death.

The relative decrease in stillbirth risk with high levels of exposure in the first trimester of pregnancy, when analyses were limited to birth residences within a 5 km radius of a monitor (data displayed in Table 4), is in contrast to the direction of effect with high exposure later in pregnancy. The biologic explanation for this difference in stillbirth risk by timing of high PM_2.5_ exposure in pregnancy is more difficult to postulate. These findings could be influenced by our study design, as only pregnancies progressing past 20 weeks were reliably captured as a birth in the vital statistics birth records, and included in the comparisons of stillbirth versus live birth risk. If high PM_2.5_ exposure in the first and early second trimester had a robust effect on some particularly vulnerable early pregnancies, resulting in miscarriage, this may have resulted in an underrepresentation of pregnancies vulnerable to PM_2.5_ exposure with high levels represented in the first trimester in this study. This could have biased risk estimates in those early pregnancy periods toward the null, or even appear protective. An additional hypothesis is there may be underlying pathophysiologic mechanisms by which an early inflammatory stimulus or hypercoagulable state might be protective later against pregnancy complications or other exposures that are known to lead to stillbirth.

### Conclusion

Although the risk increase associated with exposure to high levels of PM_2.5_ in the air is modest, the potential impact on overall stillbirth rates is robust as all pregnant women are potentially at risk. This may contribute to the higher stillbirth rates in Ohio compared to other states in the US, especially in urban areas. Based on findings from this study, we estimate the attributable risk of exposure to high levels of airborne PM_2.5_ pollution to be 0.22%. Otherwise stated, 2.2 stillbirths per 1000 may be attributed to increased exposure to PM_2.5_ in Ohio in those with high PM_2.5_ exposure. Assuming 10% of population was exposed to high particle pollution, the population attributable risk percentage of high exposure is 4% for stillbirth. These findings may indicate that improving air quality in the US could help to address the high stillbirth rate in our country, especially as compared to many other industrialized nations. [1,2]
